# Targeting of radioactive platinum-bisphosphonate anticancer drugs to bone of high metabolic activity

**DOI:** 10.1038/s41598-020-62039-2

**Published:** 2020-04-03

**Authors:** Robin A. Nadar, Kambiz Farbod, Karlijn Codee-van der Schilden, Lukas Schlatt, Barbara Crone, Nandini Asokan, Alessandra Curci, Michael Brand, Martin Bornhaeuser, Michele Iafisco, Nicola Margiotta, Uwe Karst, Sandra Heskamp, Otto C. Boerman, Jeroen J. J. P. van den Beucken, Sander C. G. Leeuwenburgh

**Affiliations:** 1 Department of Dentistry - Regenerative Biomaterials, Radboud Institute for Molecular Life Sciences, Radboud University Medical Center, Philips van Leydenlaan 25, 6525 EX Nijmegen, The Netherlands; 2 Nuclear Research & Consultancy Group, Westerduinweg 3, 1755 LE Petten, The Netherlands; 3 Institute of Inorganic and Analytical Chemistry, University of Münster, Corrensstraße 30, 48149 Münster, Germany; 4 Center for Regenerative Therapies Dresden (CRTD), Technische Universität Dresden, Fetscherstrasse 105, 01307 Dresden, Germany; 5 Division of Hematology, Oncology and Stem Cell Transplantation, Department of Medicine I University Hospital Carl Gustav Carus, Technische Universität Dresden, Fetscherstrasse 74, 01307 Dresden, Germany; 6 Dipartimento di Chimica, Università degli Studi di Bari Aldo Moro, Via E. Orabona 4, 70125 Bari, Italy; 7 National Center for Tumor Diseases (NCT), Fetscherstrasse 74/PF 64, 01307 Dresden, Germany; 8 German Consortium for Translational Cancer Research (DKTK), DKFZ, D-69120 Heidelberg, Germany; 9 Institute of Science and Technology for Ceramics (ISTEC), National Research Council (CNR), Via Granarolo 64, 48018 Faenza, Italy; 10 Department of Radiology and Nuclear Medicine, Radboud University Medical Center, Geert Grooteplein Zuid 10, 6525 GA Nijmegen, The Netherlands

**Keywords:** Medical research, Molecular medicine, Bone cancer, Bone metastases

## Abstract

Platinum-based chemotherapeutics exhibit excellent antitumor properties. However, these drugs cause severe side effects including toxicity, drug resistance, and lack of tumor selectivity. Tumor-targeted drug delivery has demonstrated great potential to overcome these drawbacks. Herein, we aimed to design radioactive bisphosphonate-functionalized platinum (^195m^Pt-BP) complexes to confirm preferential accumulation of these Pt-based drugs in metabolically active bone. *In vitro* NMR studies revealed that release of Pt from Pt BP complexes increased with decreasing pH. Upon systemic administration to mice, Pt-BP exhibited a 4.5-fold higher affinity to bone compared to platinum complexes lacking the bone-seeking bisphosphonate moiety. These Pt-BP complexes formed less Pt-DNA adducts compared to bisphosphonate-free platinum complexes, indicating that *in vivo* release of Pt from Pt-BP complexes proceeded relatively slow. Subsequently, radioactive ^195m^Pt-BP complexes were synthesized using ^195m^Pt(NO_3_)_2_(en) as precursor and injected intravenously into mice. Specific accumulation of ^195m^Pt-BP was observed at skeletal sites with high metabolic activity using micro-SPECT/CT imaging. Furthermore, laser ablation-ICP-MS imaging of proximal tibia sections confirmed that ^195m^Pt BP co-localized with calcium in the trabeculae of mice tibia.

## Introduction

Most types of tumors, i.e. breast, prostate, lung, kidney, and thyroid, metastasize to bone since its physiological environment facilitates the formation and growth of cancer cells^1,2^. These metastases affect healthy bone, which then become the primary cause of mortality^3^. Distant metastases are the leading cause of death for both breast and prostate cancer patients, with 65–75% and 90% of these patients developing bone metastases in advanced stages, respectively^4,5^. Bone metastases are often associated with accelerated bone resorption leading to complications such as skeletal-related events (SREs), bone pain or hypercalcemia^6,7^. Unfortunately, current treatments for bone metastases are limited. Bisphosphonates (BP) and denosumab are most commonly used for palliative treatment to prevent or limit SREs^8^. Although such treatments inhibit osteoclast activity and prevent the progression of metastases, they do not kill cancer cells effectively and do not improve the quality of life of patients substantially^9^. Consequently, the development of effective therapies to treat bone metastases remains a major clinical challenge.

Cancer cells modulate the bone microenvironment supporting tumor growth and accelerating tumor progression^10^. The elimination of cancer cells from bone metastases is crucial for treatment efficacy, which requires precise and efficient delivery of antitumor therapeutics to bone metastases. Clinically, bone-targeting radiopharmaceuticals such as ^153^Sm-ethylenediaminetetramethylenephosphonate and ^89^Sr-chloride are widely applied β-emitters for bone palliation^11^. Currently, ^223^Ra-dichloride is approved as a bone-seeking calcium-mimetic radiopharmaceutical for treatment of metastatic castration-resistant prostate cancer patients by emitting α-particles^12^. Moreover, clinical trials have been performed to combine ^223^Ra treatment with additional chemotherapy to enhance therapeutic efficacy^13,14^. Generally, the combination of chemotherapy with radionuclide therapy may give rise to a radiosensitizing effect where a chemotherapeutic component enhances tumor cell radiation sensitivity^15,16^.

Among various types of radiosensitizers, platinum-based (Pt) drugs belong to a class of radiosensitizers that influence the nature or repair of DNA damage^17^. Moreover, Pt-based drugs have also been investigated as radiotherapeutics by incorporation of radioactive platinum isotopes^18–20^. The incorporation of radioactive Pt atoms further enhances the therapeutic properties of Pt-based drugs^21,22^. Notably, radioisotope ^195m^Pt with a half-life of 4 days, is considered to be the most suitable Pt isotope for radionuclide therapy and medical imaging, since it is a prolific Auger electron emitter and emits a 99 keV gamma for SPECT (single-photon emission computed tomography) imaging, respectively^23,24^. Therefore, targeted delivery of Pt-based chemotherapeutics comprising ^195m^Pt could potentially increase the therapeutic efficacy of Pt-based drugs. Such targeted theranostic approach for treatment of bone metastases has the potential for precision medicine to “see and treat bone metastases”^25^.

Among the limited number of studies that evaluated the therapeutic properties of ^195m^Pt, Bodnar *et al*. demonstrated that inhibition of the growth of Ehrlich solid carcinoma by ^195m^Pt-cisplatin was 30% more effective compared to non-radioactive cisplatin^23^. Nevertheless, Pt based drugs are known to induce systemic toxicity, while the lack of tumor specificity restricts their application for long-term treatment^26,27^. Consequently, tumor-targeted delivery of Pt has strong potential to overcome the drawbacks of the currently applied Pt-based drugs^28^. Margiotta *et al*. formulated various anticancer Pt-bisphosphonate complexes aimed at targeted delivery of Pt to bone^29–31^, since BPs accumulate in bone of high metabolic activity^32^. This feature might potentially enable targeted delivery of Pt-based chemotherapeutics to bone metastases in view of the high metabolic activity of bone metastases^33,34^. However, the bisphosphonate ligands of Pt-BP compounds are presented in an entirely different manner to the mineral phase of bone as compared to free bisphosphonate distribution. Therefore, we aimed to study the biodistribution of Pt-BP compounds in metabolically active bone using advanced elemental mapping and micro-SPECT imaging techniques.

To this end, we design novel radioactive Pt-BP (^195m^Pt-BP) complexes to enable targeted delivery of ^195m^Pt to bone of high metabolic activity. First, we confirm the *in vivo* bone-seeking and Pt-DNA adduct-forming capacity of Pt-BP complexes 24 h post-administration using high-resolution inductively coupled plasma-mass spectrometry (ICP-MS). Second, we monitor the long-term stability and bone-seeking capacity of ^195m^Pt-BP over a 7-day period using preclinical micro-SPECT/CT imaging in mice. Finally, we show the spatiotemporal distribution of ^195m^Pt BP within the mice tibia using laser ablation ICP-MS imaging^35^.

## Results

### Pt-BP favors bone-specific delivery of Pt with reduced Pt-DNA adduct formation

**Figure 1:**
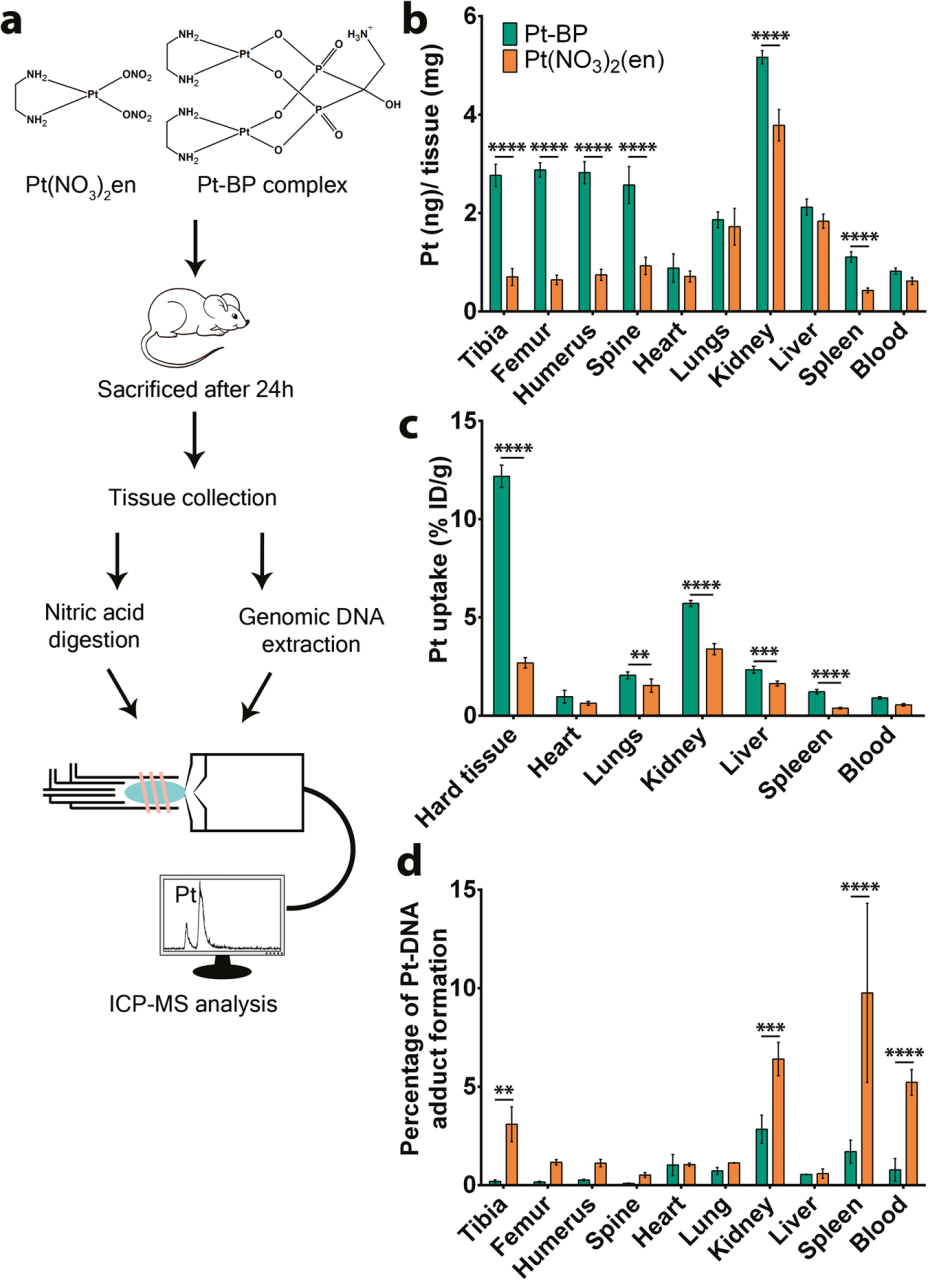
Biodistribution profile of Pt-BP and Pt(NO_3_)_2_(en) *in vivo*. (**A**) Schematic representation of Pt biodistribution study in C57Bl/6N mice. Pt-BP or Pt(NO_3_)_2_(en) compounds were administered intravenously in mice (2.5 mM Pt concentration) followed by sacrifice after 24 h. Hard tissues (tibia, femur, humerus, spine), soft tissues (heart, lungs, kidney, liver, spleen) and blood were collected. Collected tissues were subjected to nitric acid digestion and genomic DNA extraction for Pt quantification using ICP-MS and high-resolution ICP-MS, respectively. **(B)** Pt concentration in different tissues was normalized for specific tissue weight 24 h after injection. Data from 5 mice per group are represented as ng Pt/mg tissue ± SD. **(C)** Percentage of Pt uptake in different tissues 24 h after injection. Data from 5 mice per group are represented as % I.D./g ± SD of specific tissue type. **(D)** Percentage of Pt-DNA adduct formation relative to total Pt uptake in specific tissue as quantified by High Resolution-ICP-MS. Data from 4–5 mice per group are presented. **P < 0.01; ***P < 0.001; ****P < 0.0001 as determined by two-way ANOVA with a Bonferroni (multiple comparisons) post-hoc test.

We comparatively evaluated the bone-seeking properties of Pt-BP and its bisphosphonate-free precursor Pt(NO_3_)_2_(en) by quantitatively analyzing Pt biodistribution using Inductively Coupled Plasma-Mass Spectrometry (ICP-MS) in different tissues (e.g. tibia, femur, humerus, spine, blood, heart, lung, kidney, liver, and spleen) as shown in Fig. 1A. To this end, we intravenously injected sterile saline solutions containing Pt-BP or Pt(NO_3_)_2_(en) with identical Pt concentrations into the tail vein of mice, which were euthanized 24 h after injection. The dissected tissues were weighed and digested in 65% nitric acid for subsequent ICP-MS analysis of Pt content. Figure 1B shows the Pt concentrations in different tissues 24 h after injection, normalized for specific tissue weight. Pt-BP clearly showed bone-seeking properties as evidenced by higher amounts of Pt accumulation (~3 ng Pt/mg tissue) in hard tissue (bone) compared to BP-free control Pt(NO_3_)_2_(en) complex (~1 ng Pt/mg tissue).

The specificity of the two different Pt-species towards bone tissue relative to other soft tissues is depicted in Fig. 1C as the percentage of injected dose per gram of tissue (%ID/g). Higher uptake of the Pt-BP was observed in hard tissue (12.18 ± 0.56%ID/g) compared to excretory organs such as the kidney (5.70 ± 0.15%ID/g), liver (2.34 ± 0.17%ID/g) and spleen (1.22 ± 0.12%ID/g). On the other hand, Pt(NO_3_)_2_(en) showed higher uptake in kidney (3.38 ± 0.28%ID/g) compared to hard tissue (2.69 ± 0.26%ID/g), liver (1.54 ± 0.33%ID/g) and spleen (0.38 ± 0.04%ID/g). Overall, Pt-BP exhibit a 4.5-fold higher affinity for bone compared to Pt(NO_3_)_2_(en) (Fig. 1C).

The efficacy of Pt-based drugs for cancer treatment relates to the formation of Pt-DNA adducts resulting in DNA damage, which hinder mitotic processes and halt cell division. Drawbacks of the formation of Pt-DNA adducts include the high rate of DNA damage in non-target cells or healthy tissue^36^. We determined the extent of Pt-DNA adduct formation by extracting genomic DNA from different tissues and quantifying the amount of Pt using high-resolution ICP-MS. Figure 1D shows the relative Pt uptake within specific tissues leading to Pt-DNA adduct formation 24 h after injection. Pt-BP formed a low amount of Pt-DNA adducts in all tissues (<0.5%) except for the kidney (2.8%) and spleen (1.4%), which confirms the relatively low extent of DNA damage in non-targeted tissue. In contrast, the bisphosphonate-free Pt complexes showed a much higher extent of Pt-DNA adduct formation, especially in the kidneys (4.8%) and spleen (9.8%).

### ^195m^Pt-BP accumulates specifically in metabolically active bone

Radioactive ^195m^Pt-BP was synthesized using ^195m^Pt(NO_3_)_2_(en) as the precursor for ^195m^Pt. To compare the biodistribution of ^195m^Pt-BP with the precursor ^195m^Pt(NO_3_)_2_(en), a dose of 11.2 ± 0.4 MBq ^195m^Pt was administered intravenously via the tail vein in C57BL/6N mice. Although the administered Pt dose was below the Pt toxicity dose of 6 mg/kg for mice^37^, injection of ^195m^Pt(NO_3_)_2_(en) evoked a significant body weight loss within the first 3 days post injection (p.i.), leading to euthanization on reaching a humane end point. On the other hand, no behavioral or body changes were observed for ^195m^Pt-BP treated mice.

**Figure 2:**
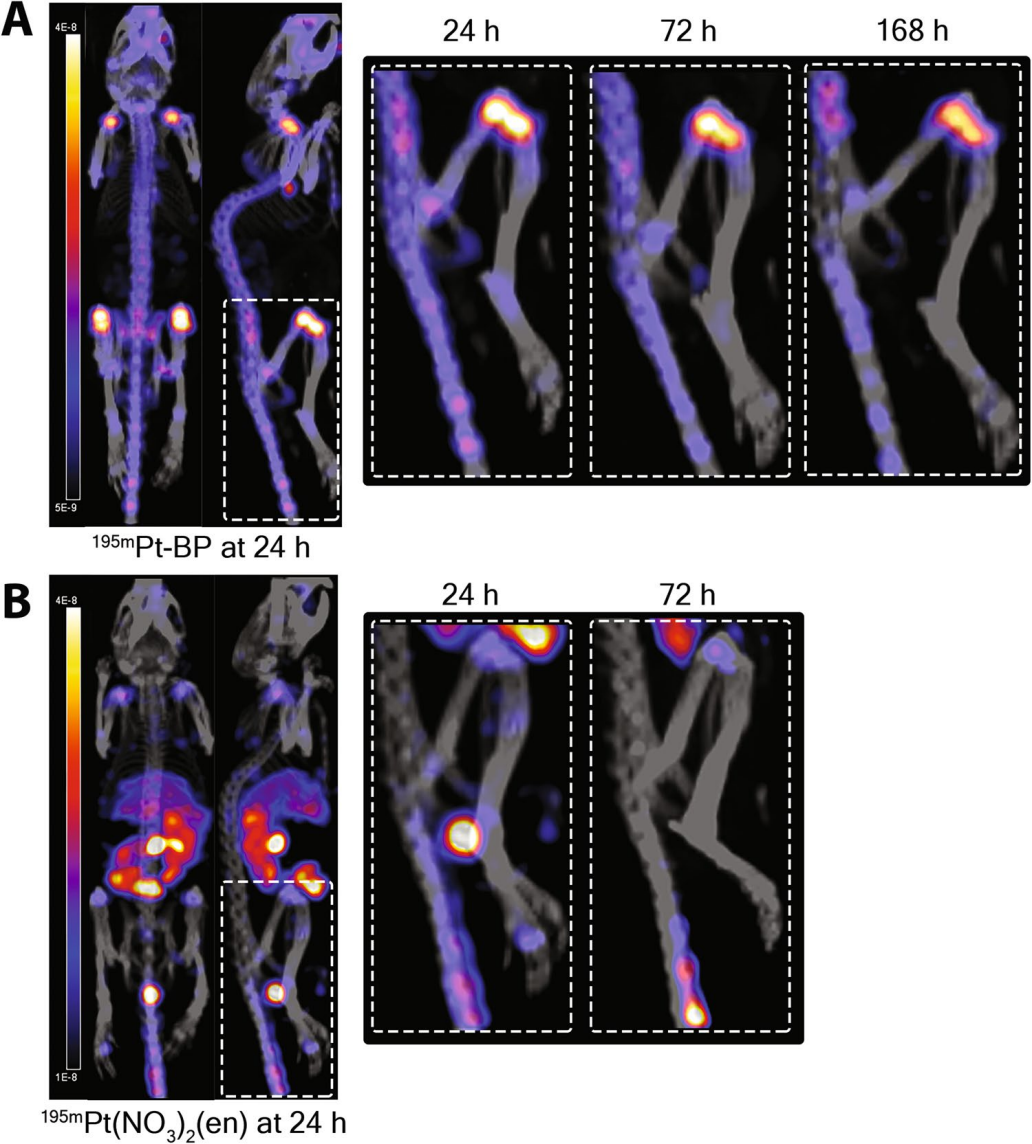
Biodistribution profile of ^195m^Pt-BP and ^195m^Pt(NO_3_)_2_(en) *in vivo*. Representative whole-body micro-SPECT/CT images of biodistribution of ^195m^Pt-BP (**A**) and ^195m^Pt(NO_3_)_2_(en) (**B**) in nude mice 24 hours after systemic administration. Rectangles show specific biodistribution of these compounds in long bones (femur-tibia) 24 h, 72 h and 168 h after systemic administration.

**Figure 3:**
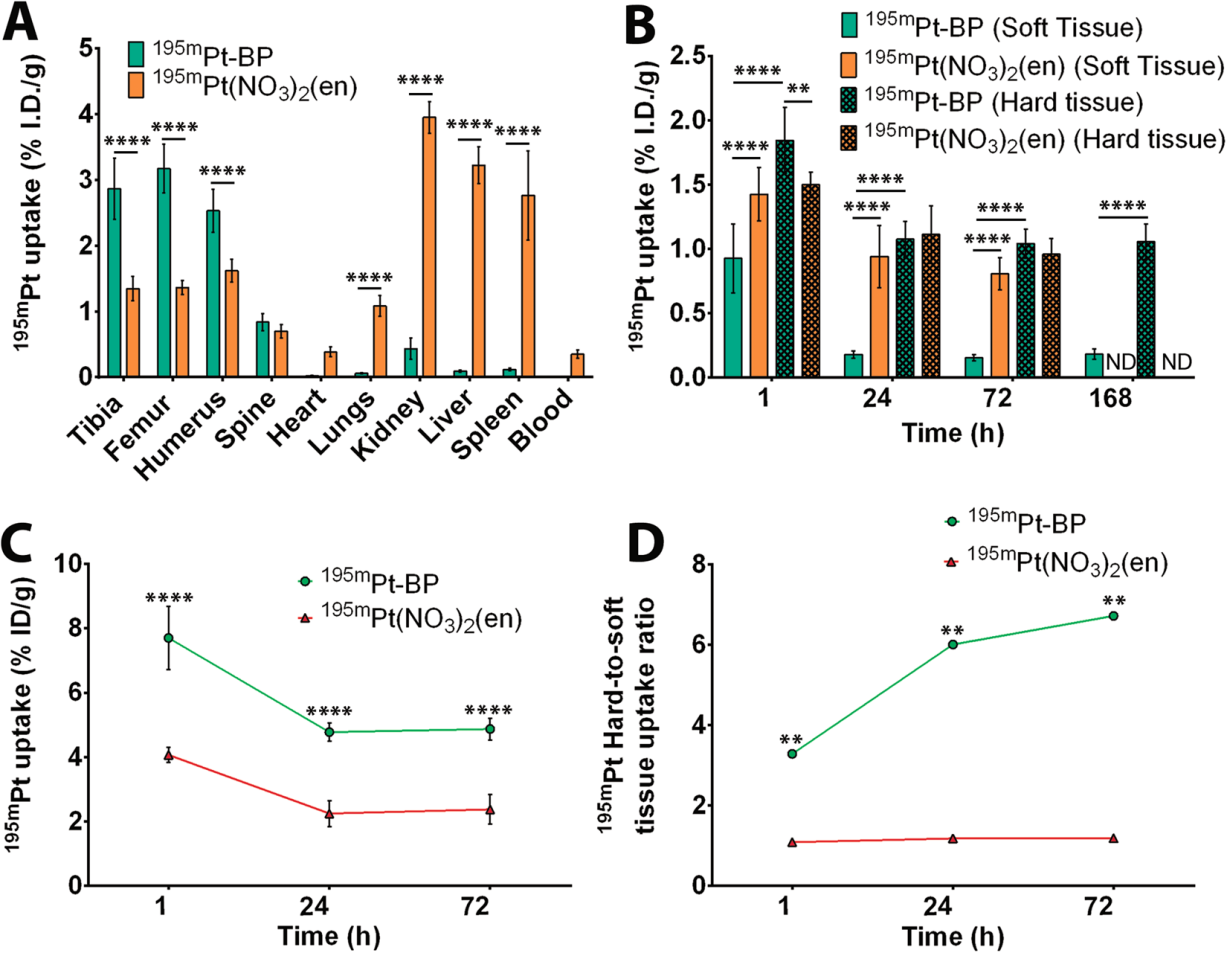
Quantification of ^195m^Pt-BP and ^195m^Pt(NO_3_)_2_(en) biodistribution *in vivo*. (**A**) Percentage of injected dose (%ID/g) of ^195m^Pt-BP (after 7 days) and ^195m^Pt(NO_3_)_2_(en) (after 3 days) in mice determined using gamma counting. (**B**) Percentage of injected dose (%ID/g) of ^195m^Pt-BP and ^195m^Pt(NO_3_)_2_(en) in mice as quantified from the micro-SPECT/CT images in soft and hard tissues. (**C**) Percentage of injected dose (%ID/g) of ^195m^Pt-BP and ^195m^Pt(NO_3_)_2_(en) in mice as quantified from the micro-SPECT/CT images in the hard tissue region of interest (ROI) with the location of the edge of the ROI contour representing 75% of maximum intensity. (**D**) ^195m^Pt hard-to-soft tissue uptake ratio excluding bladder uptake at 1 h. Data from 4–5 mice per group are presented. **P < 0.01; ****P < 0.0001 as determined by two-way ANOVA with a Bonferroni (multiple comparisons) post-hoc test. For the ratios, paired t-test was used to determine the differences among the two groups where **P < 0.01 was considered as significantly different.

Micro-SPECT/CT images were acquired 1 h – 7 days post intravenous administration of ^195m^Pt-BP (Fig. 2A). The micro-SPECT/CT scans clearly demonstrated effective targeting of ^195m^Pt-BP to growth plates in long bones, whereas ^195m^Pt(NO_3_)_2_(en) showed specific accumulation in soft tissues (Fig. 2B). Generally, growth plates are metabolically active regions in bones of young mice since they are responsible for longitudinal bone growth^38^. After excision of tissues, we quantified the uptake of radioactive ^195m^Pt-BP complexes by gamma counting (Fig. 3A). Bone-specific uptake of ^195m^Pt-BP was highest in the femur (3.1 ± 0.37%ID/g) followed by the tibia (2.86 ± 0.46%ID/g) and humerus (2.53 ± 0.33%ID/g), whereas ^19m^Pt(NO_3_)_2_(en) showed limited uptake in these bones (1.4 ± 0.15%ID/g). ^195m^Pt uptake was highest in soft tissue for ^195m^Pt(NO_3_)_2_(en) with significantly higher uptake in kidneys (3.95 ± 0.24%ID/g), liver (3.22 ± 0.28%ID/g), spleen (2.76 ± 0.67%ID/g), and lungs (1.08 ± 0.16%ID/g) compared to ^195m^Pt-BP. Conversely, ^195m^Pt-BP showed relatively low uptake in soft tissues (<0.11%ID/g), except for kidneys (0.43 ± 0.16%ID/g).

Next, we analyzed the micro-SPECT/CT images quantitatively to determine the uptake of radioactive ^195m^Pt-BP complexes. ^195m^Pt-BP showed rapid and strong uptake in bone (1.8 ± 0.25%I.D/g) at 1 h p.i., which remained constant (1.0 ± 0.12%I.D/g) from 24 h until day 7. ^195m^Pt-BP uptake was significantly lower in soft tissue than in hard tissue (0.93 ± 0.27%I.D/g at 1 h and 0.18 ± 0.03%I.D/g at 24 h). In contrast, ^195m^Pt(NO_3_)_2_(en) showed equal uptake in both hard (1.5 ± 0.1%I.D/g) and soft tissue (1.43 ± 0.21%I.D/g) at 1 h p.i., and the biodistribution remained unchanged until day 3 as shown in Fig. 3B. Subsequently, the quantification of the hot spots in bone represents a twofold increased uptake of ^195m^Pt for ^195m^Pt-BP compared to ^195m^Pt(NO_3_)_2_(en) for all time points (Fig. 3C). The hard-to-soft tissue uptake ratio of ^195m^Pt-BP increased periodically from 3.3 at 1 h, 6 at 24 h and 6.7 after 72 h. On the other hand, the hard-to-soft tissue uptake ratio of ^195m^Pt(NO_3_)_2_(en) remained almost constant at 1 at 1 h, 1.2 at 24 h and 1.2 at 72 h (Fig. 3D).

### Spatial co-localization of Pt with Ca in bone

**Figure 4:**
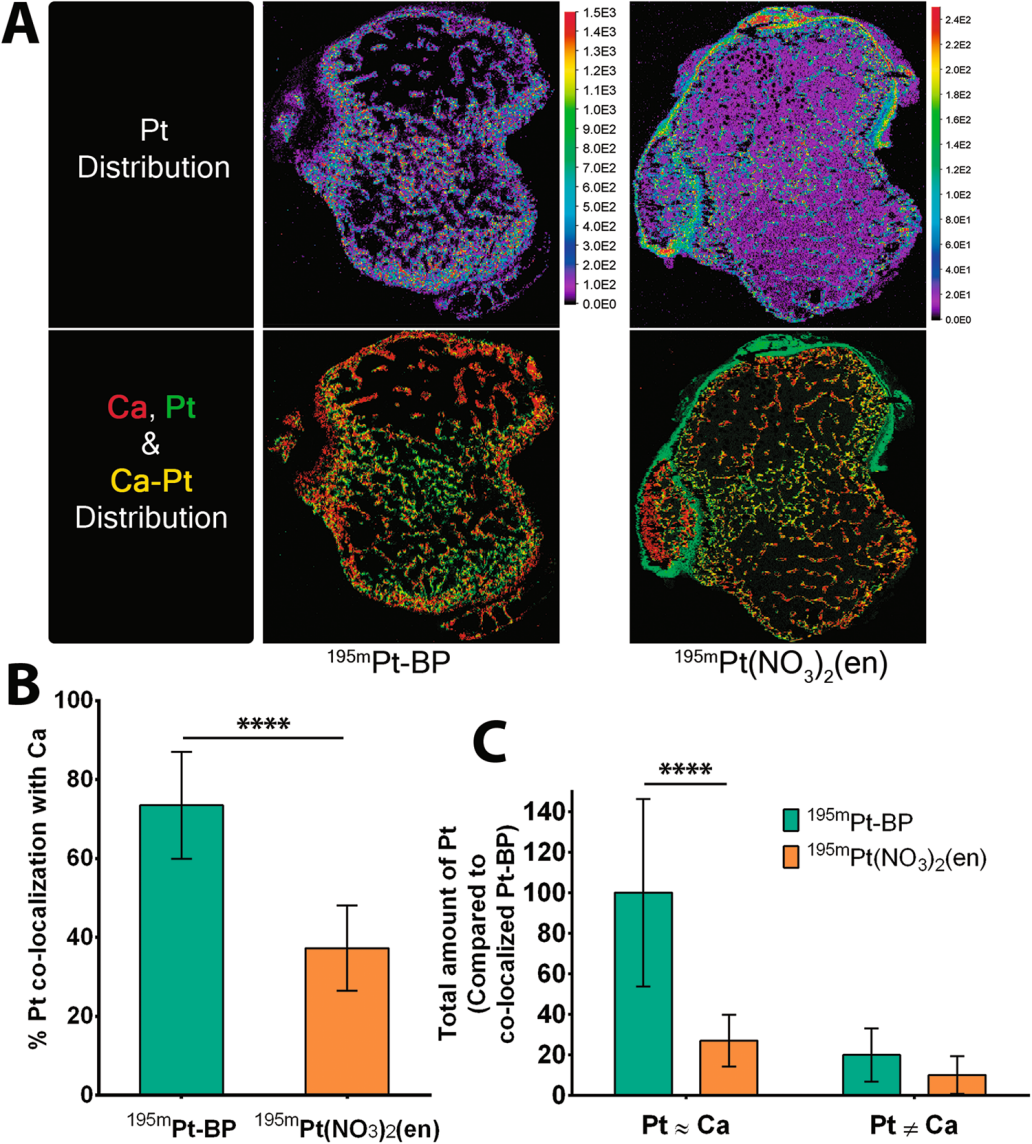
Spatial distribution of Pt in metabolically active bone. (**A**) Representative elemental mapping of platinum (Pt) in the proximal tibia of mice upon systemic administration of ^195m^Pt-BP and ^195m^Pt(NO_3_)_2_en. The bottom pictures shown an overlay of calcium (red) and platinum (green) mapping where co-localization of platinum and calcium is indicated in yellow. (**B**) Percentage of platinum co-localized with calcium in ^195m^Pt-BP and ^195m^Pt(NO_3_)_2_(en) treated mice. ****P < 0.0001, two-tailed student’s test. (**C**) Total amount of Pt co-localized with Ca (in hard tissue, Pt ≈ Ca) and Pt not co-localized with Ca (in soft tissue, Pt ≠ Ca). Data are presented from 10 sections from three tibia per group. ****P < 0.0001 as determined by two-way ANOVA with a Bonferroni (multiple comparisons) post-hoc test.

The spatial distribution of Pt as determined using Laser ablation inductively coupled plasma mass spectrometry (LA-ICP-MS) is depicted in Fig. 4. The calcium and phosphorus distribution (Supplement Fig. S1) clearly conformed to the structure of trabecular bone as observed in the dark-colored features in the microscopic image (Fig. 4A). The overlay of Ca and Pt distinctly differentiates co-localization of Pt with Ca (yellow) from Pt surrounding the trabecular structure (green). ^195m^Pt-BP showed predominant Pt uptake along the trabecular bone and inner region of the cortical bone, as reflected by an increased density of Pt co-localization with calcium. Conversely, ^195m^Pt(NO_3_)_2_(en) showed the highest Pt density in the soft tissue surrounding the cortical bone.

To quantitatively examine the co-localization of Pt with Ca, we calculated the percentage of Pt co-localized with calcium. This calculation (Fig. 4B**)** shows a co-localization of Pt with calcium of 73.5% for ^195m^Pt-BP and only 37.3% for ^195m^Pt(NO_3_)_2_(en). Relative to the extent of co-localization of Pt with Ca as observed for ^195m^Pt-BP, co-localization of ^195m^Pt(NO_3_)_2_(en) was only 27% (Fig. 4C**)**. The total amount of Pt not co-localized with Ca refers to Pt in soft tissue of the proximal tibia where 10% and 20% of Pt was detected in soft tissue for ^195m^Pt(NO_3_)_2_(en) and ^195m^Pt-BP, respectively. ^195m^Pt-BP showed almost a fourfold increased accumulation of Pt in bone compared to ^195m^Pt(NO_3_)_2_(en) as shown in Fig. 4C. These results confirm again that ^195m^Pt-BP binds specifically to the bone and effectively penetrates the trabecular structure of mouse tibiae.

## Discussion

Recent advances in imaging technologies allow early detection of malignant tissues directly in bone marrow^39,40^ or detection of indirect tumor activity related to bone remodeling^41,42^. Such early assessment of developing bone metastases would enable patient-specific bone-targeted treatment. However, treatment options for patients with bone metastases are scarce and palliative rather than curative^4,5^. Consequently, the design of novel theranostic compounds which detect and treat bone metastases in cancer patients would overcome this unmet need.

Our present work focuses on the design of radioactive and bone-seeking chemotherapeutics based on ^195m^Pt. To this end, we synthesized a Pt-BP compound comprising two ^195m^Pt moieties and a bone-seeking bisphosphonate group to target the compound specifically to metabolically active bone. This synthesis of Pt-BP was modified compared to previously reported synthesis strategies to facilitate introduction of radioactive ^195m^Pt atoms by using Pt(NO_3_)_2_(en) as Pt precursor^29,31^. ^195m^Pt(NO_3_)_2_(en) was obtained from Na_2_[^195m^PtCl_4_], which is the Pt precursor used for synthesis of radioactive cisplatin, thereby allowing for future up-scaling and clinical translation of ^195m^Pt-BP^23^.

The release of therapeutically active Pt species from Pt-BP is vital to impart the desired chemotherapeutic effects on cancer cells. Previous *in vitro* studies on Pt-BP dissociation upon release from solid hydroxyapatite nanoparticles surface confirmed that Pt-BP does not simply split into BP and Pt^31^. Pt-BP most likely releases one Pt moiety in the form of PtCl_2_(en) or related species, whereas the other Pt is sequestered with the BP molecule due to the coordination with the free amino group as present in Pt-BP. This observation is further supported by similar results obtained upon releasing Pt-BP from silica xerogels^29^.

To understand Pt release behavior *in vivo*, we investigated Pt-BP dissociation in near-physiological (pH 7.4) and acidic conditions (pH 5.25) (Supplement Fig. S2). Pt-BP underwent hydrolysis into a mononuclear Pt-n-BP derivative at both pH values, characterized by faster release of Pt at pH 5.25 than at pH 7.4. At physiological pH, Pt-BP forms new species with asymmetric bisphosphonate groups, which results most likely from displacement of one coordinated oxygen atom of the bridging bisphosphonate ligand by either a chloride (120 mM concentration) or bisphosphonate-bound amino group^43^. Besides, a signal belonging to an as yet unknown species appeared at 15.32 ppm at pH 5.25. As expected, the formation of unsymmetrical species having the amino group coordinated to the Pt atom is not favored at acidic pH, since this moiety is protonated and hence not available for coordination to the metal.

To investigate the bone-seeking ability of Pt-BP, the bisphosphonate-free compound Pt(NO_3_)_2_(en) was selected as control, since this compound was also used as precursor for the synthesis of Pt-BP. The primary reason to select Pt(en)(NO_3_)_2_ as control was that, as demonstrated by NMR on the cold Pt-BP complex, ^195m^Pt-BP releases ^195m^Pt(en)(OH_2_)_2_]^2+^, that undergoes anation reactions in physiological-like conditions. The same fate occurs also for ^195m^Pt(en)(NO_3_)_2_. In order to understand if the ^195m^Pt-species released by hydrolysis of ^195m^Pt-BP were distributed in tissues different from bone, we used the Pt-species released. Moreover, since ^195m^Pt(en)(NO_3_)_2_ was also the precursor used by the supplier (NRG) of radioactive ^195m^Pt in the synthesis of ^195m^Pt-BP, our choice allowed to reduce costs and time for the synthesis of radioactive ^195m^Pt-BP compounds. Generally, we hypothesized that the Pt(NO_3_)_2_(en) control compound lacked bone-seeking properties due to the absence of a mineral-binding moiety. Our *in vivo* biodistribution studies showed that Pt-BP compounds delivered Pt specifically to the bone. Although Pt uptake was specifically observed in hard tissues for Pt-BP compounds, this Pt did not form many Pt-DNA adducts after 24 h. These results corroborate the slow release of Pt from Pt-BP compounds observed *in vitro* at physiological pH. In contrast, Pt(NO_3_)_2_(en) showed increased Pt-DNA adduct formation in kidneys and the spleen, which are well-known off-target organs for Pt-based drugs^44^. On the contrary, the passive process of Pt-DNA adduct formation upon slow release of Pt from Pt-BP might be beneficial by diminishing undesired off-target effects.

The biodistribution of radioactive ^195m^Pt-BP and ^195m^Pt(NO_3_)_2_(en) compounds revealed a pattern similar to our previous observations using cold Pt-based therapeutics. The extended follow-up period of 7 days confirmed specific accumulation of ^195m^Pt-BP within bone only. In contrast, ^195m^Pt(NO_3_)_2_(en) specifically accumulated in kidney, liver, and spleen, thereby affecting the excretory organs and resulting in weight losses of more than 20%, which was set as humane end point. Previously, several studies have shown that cisplatin-treated mice (maximum tolerated dose of 6 mg/Kg) undergo rapid weight loss (~10%) followed by recovery within a week^37^. However, ^195m^Pt(NO_3_)_2_(en) administration resulted in more pronounced off-target effects, most likely caused by the additional radiotherapeutic properties of ^195m^Pt. The long retention time of ^195m^Pt in soft tissues as observed for ^195m^Pt(NO_3_)_2_(en) might increase radiation-induced damage caused by Auger electrons and gamma irradiation. These side-effects were not observed for ^195m^Pt-BP due to a combination of high bone-targeting efficacy and rapid excretion from soft tissues. Aalbersberg *et al*. recently demonstrated the feasibility of applying ^195m^Pt-based micro-SPECT imaging in small animals^24^. Herein, we show noninvasive detection of ^195m^Pt in bones exhibiting high metabolic activity after systemic administration of ^195m^Pt-BP. The trabecular tissue near the marrow cavity is the most metabolically active region within bones, the activity of which declines at a constant rate with aging^45^. Micro-SPECT/CT imaging with ^195m^Pt-BP showed specific accumulation of ^195m^Pt in the trabecular part of the tibia, femur, and humerus of mice. Although whole-body radioactivity in ^195m^Pt-BP treated mice was just 2%ID on day 7 (Supplement Fig. S3), the half-life of ^195m^Pt (t_1/2_ = 4 days)^23,24^ enabled quantitative, high resolution SPECT imaging. We further demonstrated ^195m^Pt-BP specificity towards skeletal sites by an increasing hard-to-soft tissue contrast from 1 h until day 3. Moreover, LA-ICP-MS imaging confirmed Pt co-localization with calcium within the trabecular region of the tibia of mice. In contrast, LA-ICP-MS imaging of the Pt distribution in ^195m^Pt(NO_3_)_2_(en)-treated mice showed preferential uptake in soft tissues (bone marrow and blood vessels) surrounding the trabecular bone. Thus, LA-ICP-MS imaging indicates that ^195m^Pt(NO_3_)_2_(en) uptake in the skeletal system is mediated by a passive mechanism via blood perfusion within the vasculature of the skeletal system.

Based on our preclinical results, ^195m^Pt-BP offers several advantages as a theranostic agent for treatment of bone metastases. First, BP moieties effectively target ^195m^Pt to bone sites with high metabolic activity. This opens up new opportunities for bone tumor-target delivery of Pt-BP compounds, since bone metastases are typically characterized by very high metabolic activity. Second, off-target effects caused by ^195m^Pt are decreased by this bone-specific targeting, rapid excretion within 24 h, and the slow release of Pt from Pt-BP. Third, ^195m^Pt-BP compounds remain stable for at least 24 h after reaching the targeted site, thereby improving the imaging quality for qualitative and quantitative diagnosis. Indeed, our results show slow release of Pt from Pt-BP *in vivo* upon attachment to hydroxyapatite mineral, which is in agreement with the *in vitro* stability of Pt-BP as observed previously using NMR. However, the main goal of systemic delivery of ^195m^Pt-BP is to deliver this ^195m^Pt (a rich source of Auger electrons) specifically to bone metastases. Considering the vigorous bone remodeling and increased bone metabolic activity in bone metastatic lesions, we assume that release of ^195m^Pt at such sites will be accelerated due to enhanced bone remodeling involving osteoclastic resorption at reduced pH. Generally, Pt-BP compounds dissociate at acidic pH, which means that release of Pt can be accelerated upon tumor-induced acidification. This hypothesis should be investigated in future studies in tumor-bearing mice. Nevertheless, further investigations are required for the translation of ^195m^Pt-BP towards clinical application.

The main goal of the study was successfully achieved, i.e. synthesis of radioactive ^195m^Pt-BP compounds which accumulated preferentially in metabolically active bone. Nevertheless, several shortcomings of the current study should be highlighted. First, the ^195m^Pt precursor ^195m^Pt(NO_3_)_2_(en) was used as the control compound since other radioactive ^195m^Pt derivatives of platinum-based drugs were not available. Second, additional therapeutic efficacy beyond the cytostatic effect of Pt may arise from Auger electrons and gamma rays emitted by ^195m^Pt, which we will investigate in a preclinical bone metastasis model in tumor-bearing mice by comparing therapeutic effects of radioactive and non-radioactive Pt-BP compounds^46^. Finally, Pt toxicity is a major concern for clinical applications of Pt-based drugs^47^. Nevertheless, we did not observe any Pt-based toxicity in ^195m^Pt-BP-treated mice over a period of 7 days. However, further long-term investigation is required to confirm adverse effect on bone biomarkers. Moreover, we addressed potential toxicity of Pt released from Pt-BP compounds in a zebrafish model, since this model provides a 500-fold higher sensitivity for early assessment of toxic effects caused by Pt-based chemotherapeutics^48,49^. As such, the zebrafish model is a highly valuable model for initial ototoxicity screening. Visual inspection of kidneys based on size and shape revealed apparent shrinkage for cisplatin controls but not for Pt-BP. In addition, no visible phenotypic effects of Pt-BP were observed in zebrafish embryos up to a concentration of 100 µM (Supplement Fig. S4). Pt-BP revealed a dose-dependent ototoxic effect on zebrafish embryos 48 h post treatment, as confirmed by staining for and quantification of lateral line neuromast hair cells (Supplement Fig. S5).

In summary, we described the synthesis and biodistribution of a bone-seeking ^195m^Pt-BP compound for active targeting of ^195m^Pt to bone sites with high metabolic activity. It should be stressed that radioactive ^195m^Pt-BP compounds with bone-seeking properties have not been reported before. Their synthesis is highly complex from a logistical perspective since access to radioactive ^195m^Pt of high specific activity is very limited worldwide. In addition, biodistribution of radioactive or inactive Pt-BP compounds has never been confirmed ***in vivo***. Our results show that for the first time using micro-SPECT imaging that radioactive ^195m^Pt-BP compounds accumulate in metabolically active bone. Finally, this accumulation was confirmed for the first time in hard tissues using elemental mapping by means of Laser ablation inductively coupled plasma mass spectrometry (LA-ICP-MS). We envision that ^195m^Pt-BP as a bone-seeking theranostic agent could meet the current clinical need to “see and treat” bone metastases in cancer patients and reduce off-target side effects of Pt-based drugs.

## Methods

### Study design

The objective of the study was to evaluate Pt-BP as potential therapeutic (Pt) or theranostic agent (^195m^Pt) to selectively deliver Pt or ^195m^Pt to metabolic active region of bone. All experiments were performed with five animals per group to reach statistical significance. Mice were randomized into different groups before administration of Pt/^195m^Pt compounds. Blinding was applied in housing. In addition, biotechnicians responsible for Pt compounds administration and monitoring of animal welfare was blinded. Image acquisition *in vivo* and *ex vivo* was performed in a non-blinded manner. Data analysis was also performed in a non-blinded manner.

### Materials

Na_2_SO_4_ and Ba(OH)_2_ were purchased from Sigma-Aldrich. 2-amino-1-hydroxyethane-1,1-diyl-bisphosphonic acid (AHBP-H_4_) was prepared following procedures reported previously^29^. Milli-Q water was used to dissolve the compounds. All other reagents were purchased from Sigma-Aldrich and used without further purification.

### Synthesis and characterization of Pt-BP complex

Pt-BP complexes with ethylenediamine(en) have been chosen as lead compounds based on previous extensive investigation^43,50^. We modified our previously reported synthesis of dinuclear bis<ethylenediamineplatinum(II) > -2-amino-1-hydroxyethane-1,1-diyl-bisphosphonate complex (Pt-BP) by using Pt(NO_3_)_2_(en) as Pt precursor instead of Pt(OSO_3_)(OH_2_)(en)^29,30,50–52^. This Pt(NO_3_)_2_(en) facilitates the synthesis of radioactive Pt-BP complexes using ^195m^Pt radioisotopes for future applications as a theranostic agent. Synthetic procedures are provided in the supplementary material.

Characterization via elemental analysis, Electrospray Ionisation-Mass Spectrometry (ESI-MS), and spectroscopic features (Supplement Figs. S6 and 7) shows consistency of the Pt-BP product with the one obtained using Pt(OSO_3_)(OH_2_)(en) as a Pt precursor^29,30^. The ^1^H-NMR spectrum of Pt-BP (Supplement Fig. S7) shows two broad singlets at 5.93 and 5.43 ppm assigned to the aminic protons of coordinated ethylenediamine^29^. The acidic conditions decelerated the exchange of the aminic protons with the deuterium of the solvent, allowing detection of the aminic protons in aqueous solution. The doublet centered at 3.34 ppm is assigned to the protons of the methylene group of the bisphosphonate. The ^31^P-NMR spectrum (Supplement Fig. S7B) of the Pt-BP in D_2_O shows a singlet with unresolved Pt satellites positioned at 37.60 ppm. This singlet can be assigned to the two phosphorus atoms of the phosphonic groups, which are magnetically equivalent and shifted at lower field (Δδ = 21 ppm) with respect to the free 2-amino-1-hydroxyethane-1,1-diyl-bisphosphonic acid (AHBP) ligand at the same pH^29^.

### Preparation of ^195m^Pt(NO_3_)_2_(en)

The ^195m^Pt was produced as previously reported^24^. The specific activity of ^195m^Pt(NO_3_)_2_(en) was 48,5 MBq/mg Pt. The radionuclide purity of ^195m^Pt(NO_3_)_2_(en) as received from NRG (Petten, The Netherlands) is reported in Supplement Table S1.

### Pt-BP pH stability assessed by NMR

^31^P NMR spectra were recorded on a Bruker Avance III 700 MHz instrument^29^. Standard pulse sequences were used for ^31^P{1H} (121.5 MHz) spectra. Chemical shifts (^31^P) were referenced to external H_3_PO_4_ (85% w/w; 0 ppm). A Crison Micro-pH meter Model 2002, equipped with Crison microcombination electrodes (5- and 3-mm diameter) and calibrated with Crison standard buffer solution at pH 4.01, 7.02, and 10.00, was used for pH measurements. The pH readings from the pH meter for D_2_O solutions are indicated as pD values and are uncorrected for the effect of deuterium on glass electrodes. The stability of Pt-BP in buffered solutions at 37 °C was assessed by ^31^P NMR spectroscopy as previously reported^53^. Pt-BP (∼4 mg) was dissolved in 0.8 mL of D_2_O containing (*i*) 50 mM 4-(2-hydroxyethyl)-1-piperazineethanesulfonic acid) (HEPES) buffer (pD = 7.4) and 120 mM NaCl or (ii) 50 mM 2-(N-morpholino)ethanesulfonic acid (MES) buffer (pD = 5.25) and 120 mM NaCl. The resulting two solutions were transferred into NMR tubes and maintained at 37 °C. ^31^P NMR spectra were recorded over a period of 15 days. The relative concentrations of the individual species in solution were deduced from integration of the ^31^P signals.

### Preparation of radioactive Pt-BP complex

The synthesis procedure was followed as described for the cold platinum-bisphosphonate complex provided in the supplementary material. The ^195m^Pt(NO_3_)_2_(en) solution was received from NRG (Petten, the Netherlands). The pH of ^195m^Pt(NO_3_)_2_(en) solution was neutralized to pH 7 using 1M NaOH and reconstituted in sterile saline solution. Characterization via elemental analysis and Electrospray Ionisation-Mass Spectrometry (ESI-MS) was not possible due to insufficient amount of residual ^195m^Pt-BP. The amount of ^195m^Pt (and thus ^195m^Pt-BP) was limited due to limited supply by NRG (Petten, the Netherlands).

### *In vivo* studies

All *in vivo* work was conducted in accordance with ISO standards and the principles set forth by the Revised Dutch Act on Animal Experimentation. The *in vivo* experiments were approved by the institutional Animal Welfare Committee of the Radboud University Medical Center (Radboudumc), Nijmegen, the Netherlands. For both the experiment, 10 male C57Bl/6N mice (Charles River), with an average weight of ~25 g and an age of approximately 6–8 weeks were housed in filter-topped cages (5 mice per cage) under non-sterile standard conditions provided with standard animal food and water ad libitum. The mice were allowed to adapt to laboratory conditions for 1 week before experimental use. The humane end point were defined as follows, body weight gain of> 20% within 10 days, no water or food intake, weight loss of >15% within 1–2 days or> 20% of normal body weight of that animal, serious circulatory or respiratory problems, behavioural changes (hyperactivity, passive, auto-mutilation), and morbidities such as impaired mobility or lethargy^52^.

### *In vivo* biodistribution of cold Pt-species and quantification of Pt accumulation

The Pt-BP and Pt(NO_3_)_2_(en) complexes were administered intravenously in the tail vein of C57BL/6N mice (n = 5 per each platinum complex). Sterile saline solution (0.9% NaCl) was used to dissolve platinum complexes. The concentration of injected Pt-BP or Pt(NO_3_)_2_(en) solutions was 2.5 mM platinum, calculated based on the maximum tolerated dose for cisplatin of 6 mg per kg body weight of the mice^37^. The mice were euthanized with CO_2_ 24 h after injection, after which blood (approximately 600 mg per mouse), liver, spleen, kidneys, heart, lungs, and bones (femur, humerus, tibia, and spine) were harvested. Approximately half of the tissues were prepared for inductively coupled plasma-mass spectrometry (ICP-MS) analysis by digestion in 65% (v/v) nitric acid at 75 °C for approximately three days until the tissues were digested completely. Each tissue was cut into three samples and the samples were weighed prior to digestion in nitric acid. The digested solutions were diluted with up to 6 ml of Milli-Q water to obtain 2% (v/v) nitric acid in order to measure platinum concentrations by ICP-MS (X series I, Thermo Electron Corporation). The detection limit for determining platinum concentration with ICP-MS was 1 ppb. The standard solutions were prepared from 1000 mg·1^−1^ platinum ICP standard Certipur (1.70341.0100, Merck) ranging from 1 ppb to 2500 ppb. The measured isotopes for platinum were ^194^Pt, ^195^Pt, ^196^Pt, and ^198^Pt. Furthermore, scandium (^45^Sc) was added as an internal standard, which was prepared with 1000 mg·1^−1^ scandium standard Certipur (1.19513.0100, Merck) to correct for matrix effects and long-term fluctuations of the measurement signal. The platinum concentrations in the various tissues were calculated relative to 1 mg of the specific tissue and represented as ng Pt/mg_tissue_. Three replicates of Pt-BP and Pt(NO_3_)_2_(en) solutions with volumes and platinum concentrations identical to the solutions injected to the mice (200 µl and 2.5 mM platinum, respectively) were also analyzed for their platinum content using ICP-MS as control. The percentage of injected dose (%ID) of Pt-BP and Pt(NO_3_)_2_(en) in each mouse tissue 24 h after injection was calculated by dividing the total amount of platinum per tissue through the total amount of platinum detected in the control solutions^52^.

### Pt-DNA adducts quantification using High Resolution ICP-MS

The remaining half of the tissues from all mice were dissected and weighed for DNA extraction. DNA was isolated using DNeasy Blood & Tissue Kit (Qiagen, USA) for soft tissue and ChargeSwitch® gDNA Plant Kit (Thermofisher, USA) for bone samples. The DNA was digested with DNase I Solution (Thermofisher, USA) and filtered using 0.2 µm acrodisc GHP before analysis for Pt content using high resolution-ICP-MS (Element2, Thermo Finnigan). The detection limit for determining the platinum concentration was 2 ng/l. The standard solutions were prepared from 1000 mg·1^−1^ platinum ICP standard Certipur (1.70341.0100, Merck) ranging from 5 ppt to 10 ppb. The selected isotopes for platinum were ^194^Pt and ^195^Pt. Furthermore, thallium (Tl) was added as an internal standard and determined at a molecular mass of 205 Da, which was prepared using a 1000 mg·1^−1^ thallium standard (CGTL-1, Inorganic Ventures) to correct for matrix effects and long-term fluctuations of the analytical signal. The Pt-DNA adduct concentration is represented as percentage of Pt involved in adduct formation to the total amount of Pt accumulated in the specific tissue^52^.

### *In vivo* biodistribution of radioactive Pt-species and micro-SPECT quantification of ^195m^Pt biodistribution

The ^195m^Pt-BP and ^195m^Pt(NO_3_)_2_(en) complexes were reconstituted in sterile saline solution (0.9% NaCl) and administered intravenously in the tail vein of C57BL/6N mice (n = 5 per each platinum complex). All mice received an intravenous injection with a dose of 11.2 ± 0.4 MBq ^195m^Pt. Immediately after injection and 1, 3, and 7 days after injection, images were acquired with U-SPECT-II/CT(MILabs) as reported previously^54,55^. Mice were scanned under general anesthesia (isoflurane/O_2_) for 15 to 60 minutes using the 1.0-mm diameter pinhole mouse high sensitivity collimator tube, followed by a CT scan (spatial resolution 160 mm, 65 kV, 615 mA) for anatomical reference. Scans were reconstructed with MILabs reconstruction software using an ordered-subset expectation maximization algorithm, with a voxel size of 0.4 mm. SPECT/CT scans were analyzed and maximum intensity projections were created using the Inveon Research Workplace software (IRW, version 4.1). A 3D volume of interest was drawn using CT threshold to differentiate soft tissue from skeletal tissue and uptake was quantified as the percentage injected dose per gram (%ID/g), assuming a tissue density of 1 g/cm^3^. The hot spot in the skeletal tissue region of interest (ROI) was chosen with the location of the edge of the ROI contour representing 75% of maximum intensity. The mice from ^195m^Pt(NO_3_)_2_(en) group were euthanized with CO_2_ after 3 days due to excessive loss of body weight, whereas mice from treated with ^195m^Pt-BP were sacrificed at the end of experiments. After euthanizing the animals, blood (approximately 600 mg per mouse), liver, spleen, kidneys, heart, lungs, and bones (femur, humerus, tibia, and spine) were harvested and analyzed with a gamma counter (1470 Wizard, Perkin Elmer).

### Laser ablation ICP MS imaging of ^195m^Pt biodistribution in mice tibia

The experimental procedure was carried out as reported previously^35^. The mice tibias were incubated in neutral buffered formaldehyde for 36 h. Later, tibias were dehydrated in ascending grades of 70% to 100% ethanol and embedded in poly(methyl methacrylate) (pMMA) resin, freshly prepared by mixing 600 mL of methyl methacrylate monomer (Acros Organics BVBA, Geel, Belgium), 60 ml dibutyl phthalate (Merck KGaA, Darmstadt, Germany) and 1.25 g perkadox^®^16 (Aldrich, Netherlands). The polymerization was followed by cutting serial horizontal sections (perpendicular to the tibia sample) of 5 µm thickness within the trabecular region of interest using an RM2155 microtome with a TC 65 blade (Leica Microsystems GmbH, Wetzlar, Germany). Microscopic images were obtained using an inverted fluorescence/bright field microscope (BZ-9000, Keyence Deutschland GmbH, Neu-Isenburg, Germany). For image recording and processing, the software BZ-II Viewer and BZ-II Analyzer were used, respectively. To calculate the percentage of platinum co-localized with calcium, the background of the image and all pixels without hard bone tissue were excluded from the calculations. A pixel is considered to be background if its calcium intensity is below 15%. The platinum concentrations of the remaining pixels were then added up and divided by the sum of the entire platinum image to yield the percentage of platinum co-localized with calcium^35^.

### Zebrafish care and handling

The zebrafish (Danio rerio) strains were kept under standard conditions (28 °C in E3 buffer) until 48 hours post fertilization (hpf) as described previously^56^. All animal experiments were conducted at larval stages before the point of independent feeding and were in agreement with the animal protection law (Tierschutzgesetz).

### Phenotypic assessment of drug-treated embryos

48 hpf wildtype embryos were separated into 6 groups (n = 20 embryos/group), i.e., control (untreated) and embryos treated with 5 µM, 10 µM, 50 µM, and 100 µM Pt-BP as well as 30 µM cisplatin. Both types of compounds were added externally to the medium and the embryos were incubated at 33 °C for the duration of the experiment. The embryos were assessed each day for phenotypic toxicity until 2 days post treatment (48 hpt) and imaged live in an Olympus MVX10 microscope.

### Ototoxicity assay

The lateral line neuromast hair cells in embryos were stained with a vital dye to analyse the loss of hair cells after Pt-BP treatment. Cisplatin was used as a control, as it was known to affect hair cells in humans. The fluorescent dye 2-[4-(dimethylamino)styryl]-N-ethylpyridinium iodide (DASPEI) [Molecular Probes, Eugene, OR] was used to stain hair cells in the neuromast as described previously^57^. Larvae were incubated in embryo medium containing 0.005% DASPEI for 15 min, anesthetized in Tricaine MS222 (10 lg/ml) for 5 min, rinsed once in fresh embryo medium, and imaged using Vertebrate automated screening technology (VAST, Union Biometrica), combined with high-speed and super-resolution Zeiss cell observer spinning disk confocal system. The quantification of number of neuromast hair cells in the treated embryos was done manually with confocal microscopy (Zeiss LSM780).

### Statistical analysis

All results except ratios are depicted as mean ± standard deviation. The statistical analyses were performed using GraphPad Prism (version 6.0) software. Two-way analysis of variance (ANOVA) with a Bonferroni (multiple comparisons) post-hoc test was used to determine the differences among the two groups. For ratios, paired t-test was used to determine the differences among the two groups. For the ototoxicity assay, one-way analysis of variance (ANOVA) was performed followed by the Dunnett’s method for multiple comparisons.

## Supplementary information

Supplementary Information.
