# Supplementary material for: Targeting of radioactive platinum-bisphosphonate anticancer drugs to bone of high metabolic activity
Source: Sci Rep. 2020 Apr 3;10:5889. doi: 10.1038/s41598-020-62039-2 (PMC7125202; doi:10.1038/s41598-020-62039-2)
Supplement: Supplementary file 1 — Supplementary Information. [file 41598_2020_62039_MOESM1_ESM.docx]

Supplementary Information

Targeting of radioactive platinum-bisphosphonate anticancer drugs to bone of high metabolic activity

*Robin A. Nadar, Kambiz Farbod, Karlijn Codee-van der Schilden, Lukas Schlatt, Barbara Crone, Nandini Asokan, Alessandra Curci, Michael Brand, Martin Bornhaeuser, Michele Iafisco, Nicola Margiotta, Uwe Karst, Sandra Heskamp, Otto C. Boerman, Jeroen J. J. P. van den Beucken and Sander C. G. Leeuwenburgh**

* Corresponding author. Email: [sander.leeuwenburgh@radboudumc.nl](mailto:sander.leeuwenburgh@radboudumc.nl)

**List of Supplementary Materials**

Pt release from Pt-BP at physiological and acidic, tumor-mimicking pH.

Methods:

Preparation and Characterization of Pt(NO_3_)_2_(en)

Preparation and Characterization of Pt-BP

Figure S1. Spatial distribution of Ca and P in metabolically active bone.

Figure S2. Synthesis and Characterization of Pt-BP complex.

Figure S3. ^195m^Pt radioactivity in mice.

Figure S4. In vivo phenotypic effects of Pt-BP in zebrafish embryos.

Figure S5. Ototoxicity caused by Pt-BP treatment of zebrafish embryos.

Figure S6. FTIR spectra of Pt-BP complex.

Figure S7. NMR characterization of Pt-BP complex.

Table S1. Characteristics of the radionuclide purity of ^195m^Pt(NO_3_)_2_(en).

**Supplementary Materials**

**Pt release from Pt-BP at physiological and acidic, tumor-mimicking pH**

In vitro investigations on the stability of Pt-BP showed that the complex is stable in neutral aqueous solution (pH 7.0, room temperature) for several months^1^, while structural changes became apparent at pH > 8.0 with the formation of new products having inequivalent phosphorous atoms (as revealed by ^31^P NMR spectroscopy). However, we did not observe release of free bisphosphonate, indicating that Pt-BP rearranges rather than decomposes in water at pH > 8.0^2^.

To investigate the stability of Pt-BP in near-physiological conditions, we utilized a medium consisting of D_2_O containing HEPES buffer (50 mM, pH = 7.4) and NaCl (120 mM) at 37 °C, in which we analyzed Pt-BP stability by ^31^P-NMR spectroscopy. In Supplement Fig. 2A at pD = 7.4 and 37 °C, Pt-BP underwent rapid hydrolysis leading to formation of several species in equilibrium in solution. A symmetric mononuclear Pt-n-BP derivative M obtained with the release of a Pt(en) moiety was evidenced by the decrease in intensity of the starting Pt-BP signal in the ^31^P spectrum (36.40 ppm; indicated with Pt-BP in Supplement Fig. 2A) with simultaneous formation of a singlet at 23.6 ppm (M in Supplement Fig. 2A). Two other asymmetrical species are visible in the ^31^P-NMR spectrum recorded after 3 h as evidenced by the two pairs of doublets falling at 28.15 and 22.98 ppm and 26.25 and 21.51 ppm (indicated by the black squares and diamonds in Supplement Fig. 2A). We tentatively assign these signals to two asymmetrical species obtained by coordination of the NH_2_ group of the dangling arm which is partially deprotonated at pD 7.4 or by coordination of a chloride ion (120 mM in the medium) with displacement of a coordinated bisphosphonate oxygen atom. No traces of the starting Pt-BP complex were observed after 24 h. Finally, the second Pt(en) moiety was released only after 96 h as confirmed by the appearance of free BP (singlet at 17.10 ppm, indicated BP in Supplement Fig. 2A).

To simulate the acidic environment in tumor tissue^3,4^, we explored the stability of Pt-BP complexes at pD = 5.25 at 37 °C by ^31^P-NMR spectroscopy (Supplement Fig. 2B). In contrast to our observations at pD 7.4, at acidic pD, where the NH_2_ group is protonated and not capable to coordinate to the Pt atom, the hydrolysis of Pt-BP leads to the formation of only the monomeric Pt-n-BP derivative (M in Supplement Fig. 2B). However, the formation of M was slower at acidic pD, while its hydrolysis, with concomitant formation of free bisphosphonate (BP), was faster than at pD 7.4 since Pt-BP and BP were detected after 48 h (Supplement Fig. 2B). Thus, fast release of a single Pt(en) moiety Pt-BP was observed at pD 7.4 within 24 h, whereas faster release of both Pt(en) moieties were observed at pD 5.5 after 48 h.

**Methods**

## *Preparation and Characterization of Pt(NO_3_)_2_(en)*: Pt(NO_3_)_2_(en) (en = ethylenediamine) was synthesized according to a previously reported procedure^5,6^. Briefly, K_2_PtCl_4_ (100 mg, 0.241 mmol) was dissolved in Milli-Q water (2.5 ml) and stirred at room temperature. Upon complete dissolution, the solution was filtered using a sintered glass filter to remove undissolved platinum. Subsequently, potassium iodide (KI; 239.9 mg, 1.445 mmol) was added to the filtrate at room temperature, agitating for approximately 5 min. 16.08 µl (0.441 mmol) of ethylenediamine diluted in 400 µl of Milli-Q water was then added slowly and drop-wise under constant magnetic stirring and left at room temperature for approximately 2 h. The obtained yellow precipitate was filtered with a sintered glass filter. The solid phase was washed with cold Milli-Q water, absolute ethanol, and finally with diethyl ether, followed by drying under vacuum. The obtained PtI_2_(en) (58.6 mg, 0.115 mmol) was suspended in 16.4 ml of Milli-Q water at 55 °C in the dark and treated with AgNO_3_ (39.12 mg, 0.23 mmol) previously dissolved in 131 µl Milli-Q water protected from light. The mixture was stirred for approximately 4 h in the dark. The flask was then cooled down to room temperature prior to filtration through a plug of Celite^®^ to remove AgI. The filtrate was dried using a rotary evaporator (at 40 °C) followed by vacuum drying. Electrospray Ionisation-Mass Spectrometry (ESI-MS) was carried out to determine the molecular mass of the obtained Pt(NO_3_)_2_(en) using an electrospray interface and ion trap mass spectrometer (1100 Series LC/MSD Trap system Agilent, Palo Alto, CA): *Anal. Calc.* for Pt(NO_3_)_2_(en): (C_2_H_8_N_2_O_6_Pt, M_w_ = 351.17 g·mol^-1^). Spectroscopic characterization of the complex with Attenuated Total Reflectance-Fourier Transform Infrared spectroscopy (ATR-FTIR; Spectrum Two^™^, Perkin Elmer) was consistent with literature data (data not shown).

***Preparation and Characterization of Pt-BP***

A solution containing 2-amino-1-hydroxyethane-1,1-diyl-bisphosphonic acid (AHBP-H_4_) (3.33 mg, 0.015 mmol; in 2.2 ml Milli-Q water) was maintained at 40 °C and then treated with Ba(OH)_2_^.^8H_2_O (4.97 mg, 0.01575 mmol). The mixture was left under stirring at 40 °C and then treated with a solution of Pt(NO_3_)_2_(en) (12.5 mg, 0.033 mmol) and Na_2_SO_4_ (2.24 mg, 0.01575 mmol) in 1.7 ml Milli-Q water. The obtained suspension was subsequently added drop-wise to the main vial containing AHBP-H_4_ and Ba(OH)_2_^.^8H_2_O agitating at 40 °C. A white suspension was formed which was left overnight under constant stirring at 40 °C. Subsequently, the suspension was cooled down for approximately 1 h in an ice bath to facilitate the precipitation of BaSO_4_ prior to filtration through a plug of Celite^®^. The volume of the filtrate, which contained the final product, was reduced using a rotary evaporator (at 40 °C) and the pH of the concentrated filtrate was brought to ~1 using H_2_SO_4_ (95–97%). Addition of methanol induced the precipitation of the desired product as a white precipitate that was filtered and washed with methanol and diethyl ether, and subsequently dried under vacuum. Elemental analyses were carried out using a Hewlett Packard 185 C and N analyzer. ESI-MS was carried out to determine the molecular weight of the obtained Pt-BP using an electrospray interface and ion trap mass spectrometer. *Anal. Calc.* for [{Pt(en)}_2_(μ-AHBP-H_2_)](HSO_4_)⋅3H_2_O (C_6_H_29_N_5_O_14_P_2_Pt_2_S, M_w_ = 879.4 g·mol^-1^): C, 8.19%; N, 7.96%. Found: C, 7.99%; N, 7.45%. Spectroscopic characterization of the Pt-BP was carried out using ^1^H and ^13^P Nuclear Magnetic Resonance (NMR) spectroscopy and ATR-FTIR^5^.

**
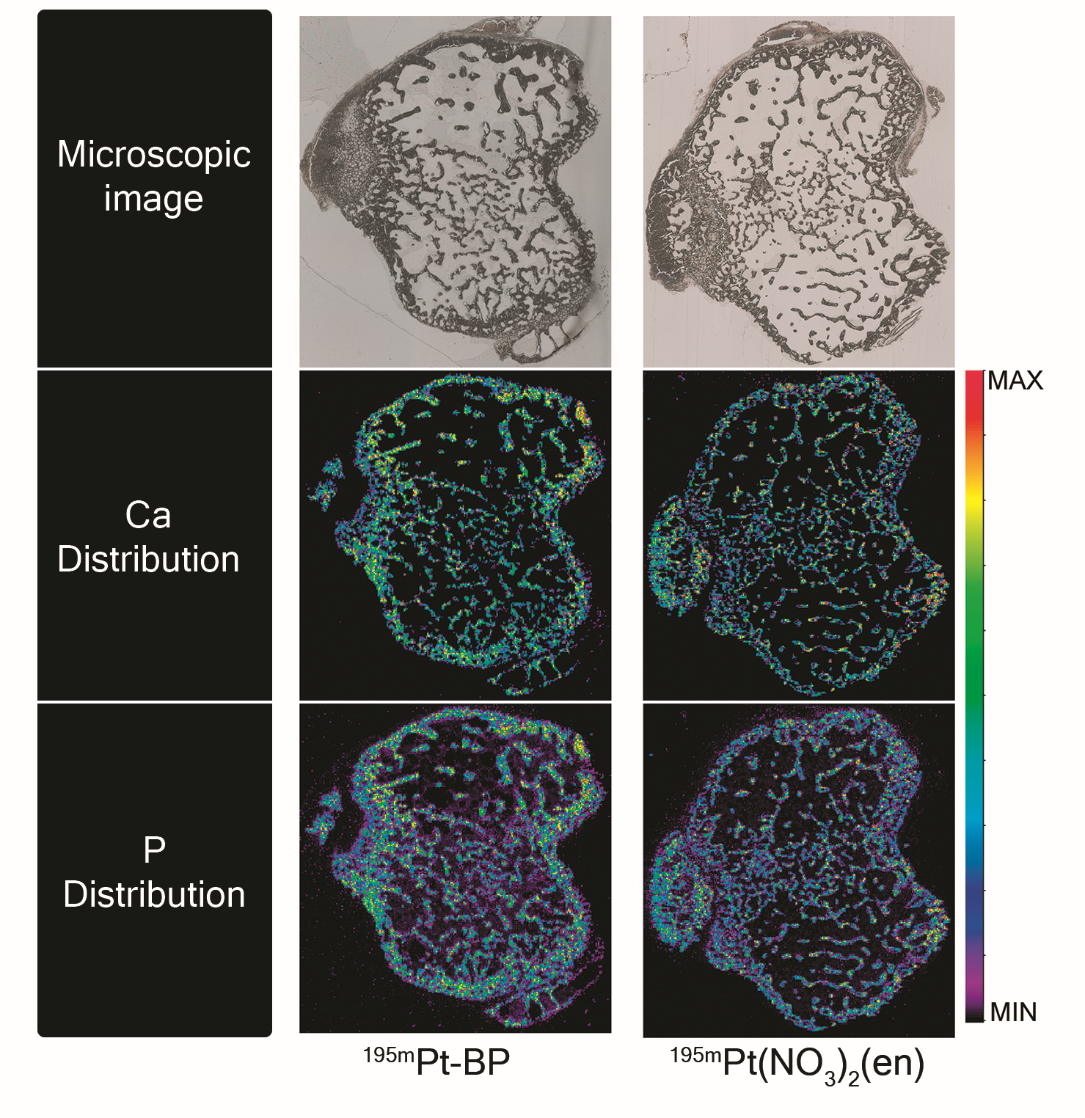
**

**Figure S1.** Spatial distribution of Ca and P in metabolically active bone. Representative elemental mapping of calcium (Ca) and phosphor (P) in the proximal tibia of mice.

**
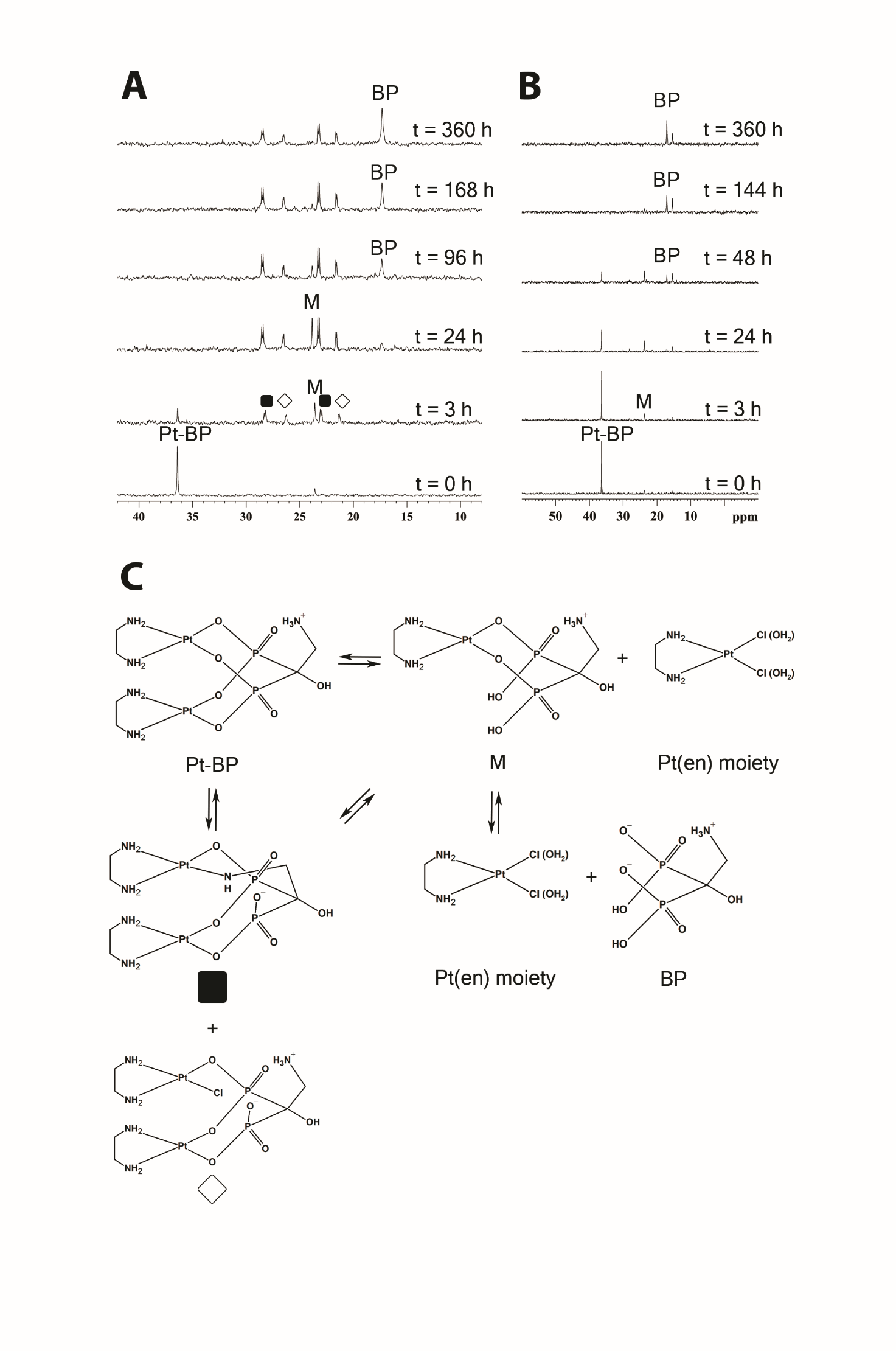
**

**Figure S2.** NMR characterization of Pt-BP complex. A) ^31^P-NMR (121.5 MHz) spectra of Pt-BP at diﬀerent time points in near-physiological conditions (D_2_O, HEPES buﬀer 50 mM, pD = 7.4, 120 mM NaCl, 37 °C). B) ^31^P-NMR (121.5 MHz) spectra of Pt-BP at diﬀerent time points at pD = 5.25 (D_2_O, MES buﬀer 50 mM, 120 mM NaCl, 37 °C). C) Schematic representation of Pt speciation from Pt-BP as assessed by ^31^P-NMR spectroscopy. Pt-BP undergoes hydrolysis to release a Pt(en) moiety and a symmetric monomeric Pt-n-BP derivative (M). The second Pt(en) moiety is released by the hydrolysis of M with the release of free BP. At intermediate time points, Pt-BP is also in equilibrium with new species with asymmetric bisphosphonate giving two signals in the ^31^P-NMR spectrum and labeled with the black square and diamond symbols.

**
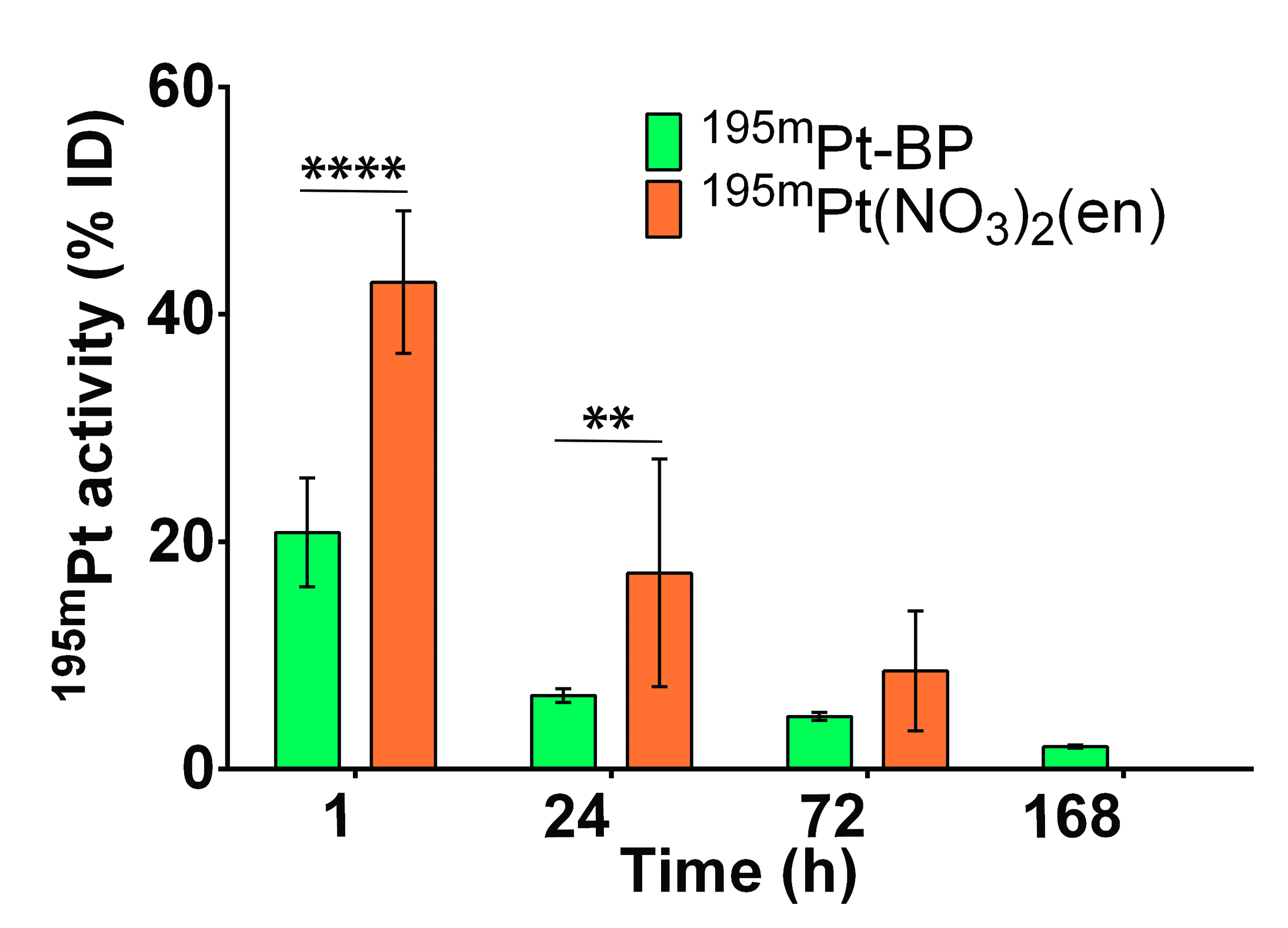
**

**Figure S3**. ^195m^Pt radioactivity in mice. Percentage of injected dose (%ID) of ^195m^Pt-BP and ^195m^Pt(NO_3_)_2_(en) in mice, measured using an ionization chamber. ** P < 0.01; ****P < 0.0001 as determined by two-way ANOVA with a Bonferroni (multiple comparisons) post-hoc test.


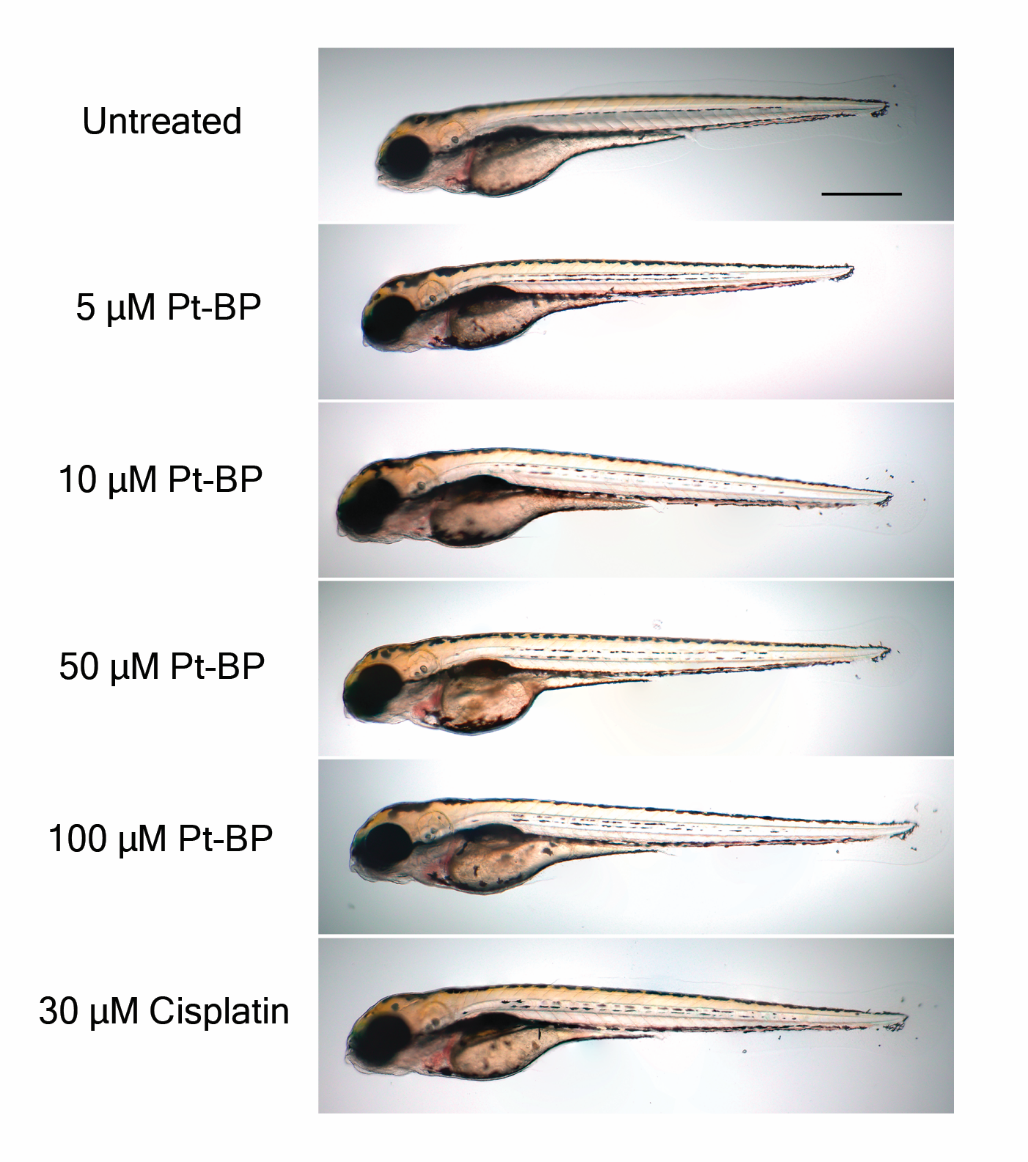


**Figure S4.** In vivo phenotypic effects of Pt-BP in zebrafish embryos. Representative images of embryos treated with different concentrations of Pt-BP. Until 2 days post treatment (dpt), Pt-BP-treated embryos did not show any phenotypic abnormality in comparison to untreated and cisplatin-treated embryos. Scale bar: 50 µm.


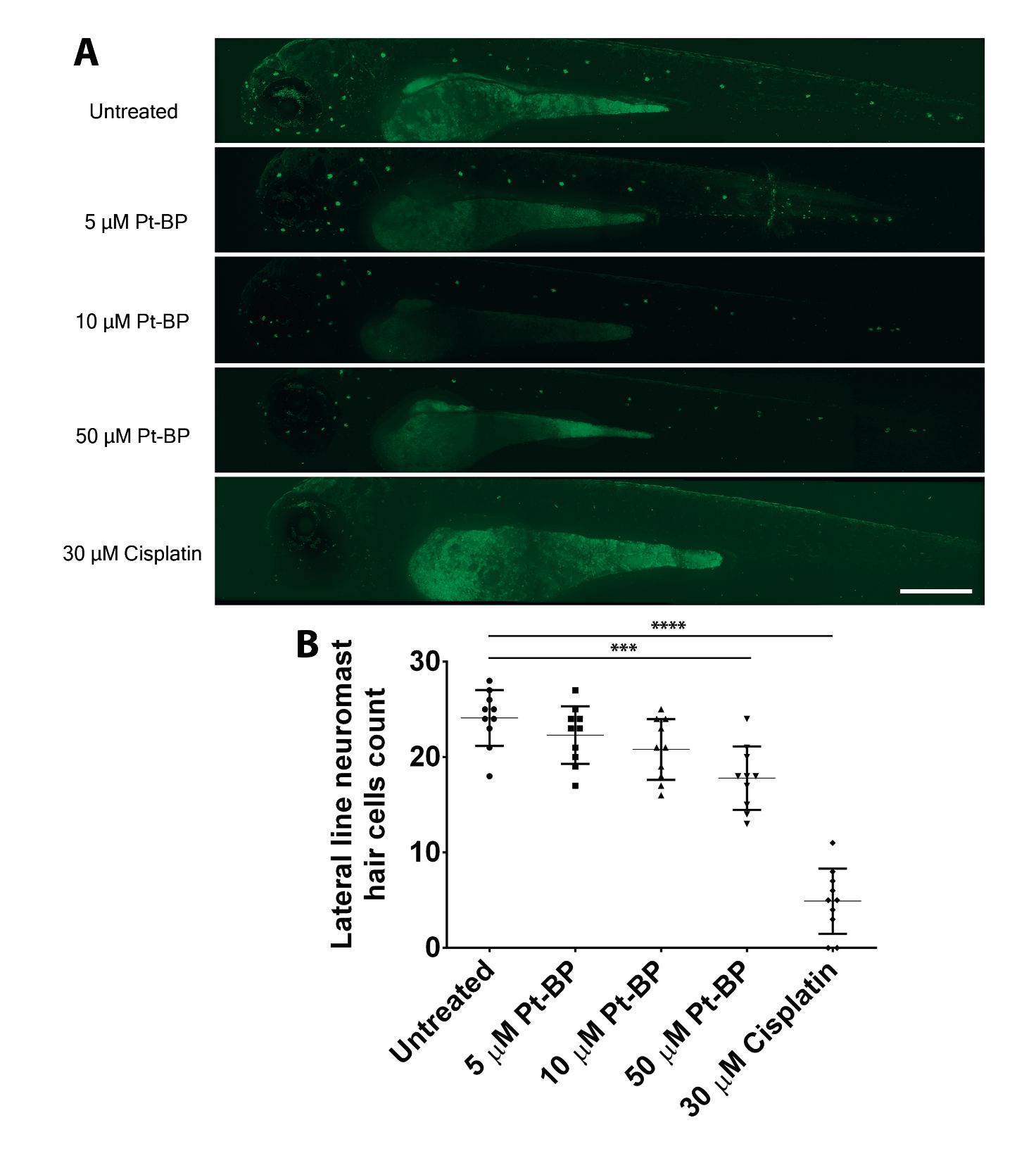


**Figure S5.** Ototoxicity caused by Pt-BP treatment of zebrafish embryos. A) Embryos were treated with Pt-BP (5, 10, and 50 µM) and cisplatin (positive control, 30 µM) and lateral line neuromasts were stained using DASPEI. B) Quantification of the lateral line neuromast cells stained by DASPEI after 48 h Pt-BP and Cisplatin treatments. ***P < 0.001; ****P < 0.0001 by one-way ANOVA, followed by Dunnett’s method for multiple comparisons. Scale bar: 50 µm.

**
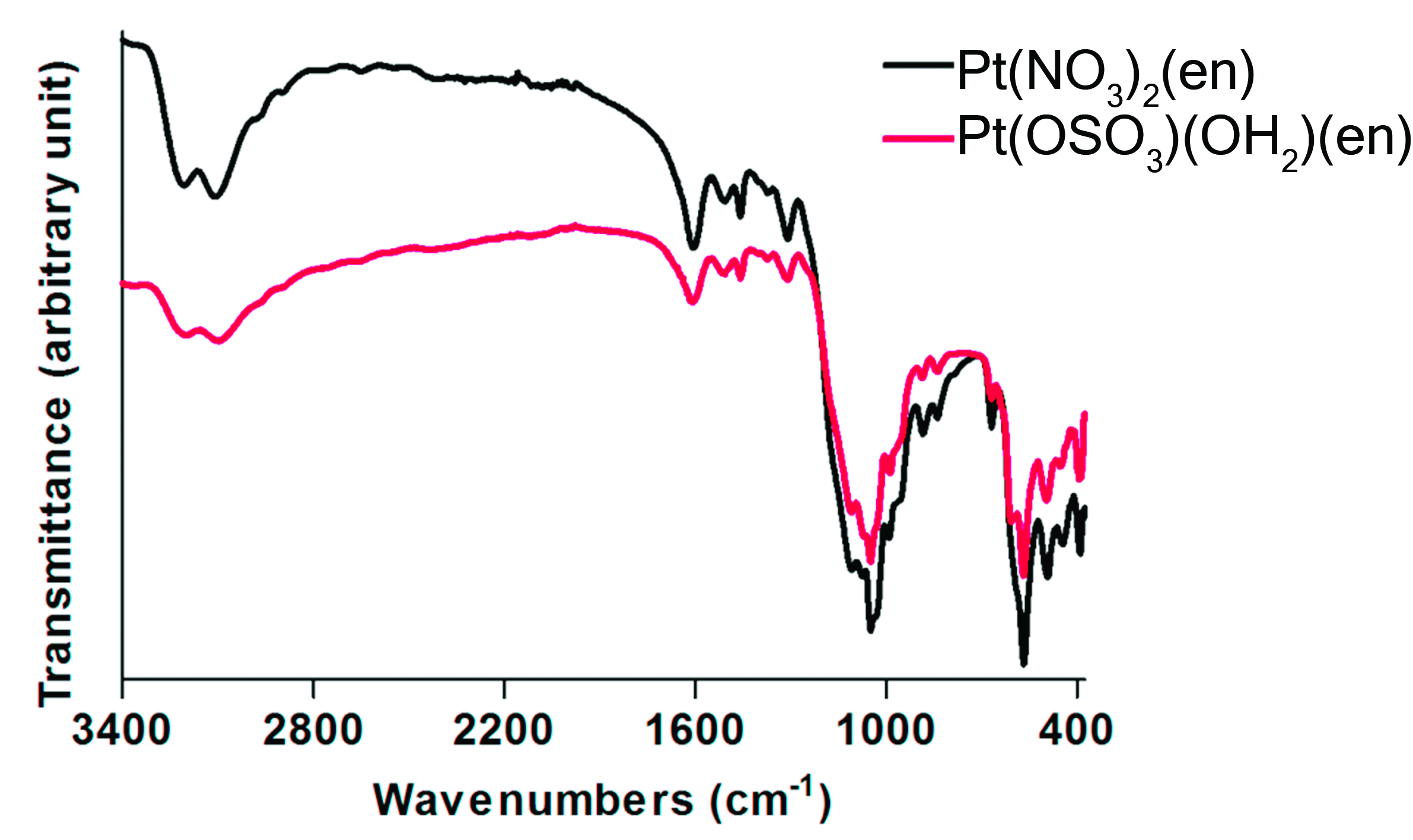
**

**Figure S6.** FTIR spectra of Pt-BP complex. Pt-BP complex infrared spectra prepared using Pt(NO_3_)_2_(en) as the precursor versus Pt-BP complex prepared using Pt(OSO_3_)(OH_2_)(en) as the precursor.

**
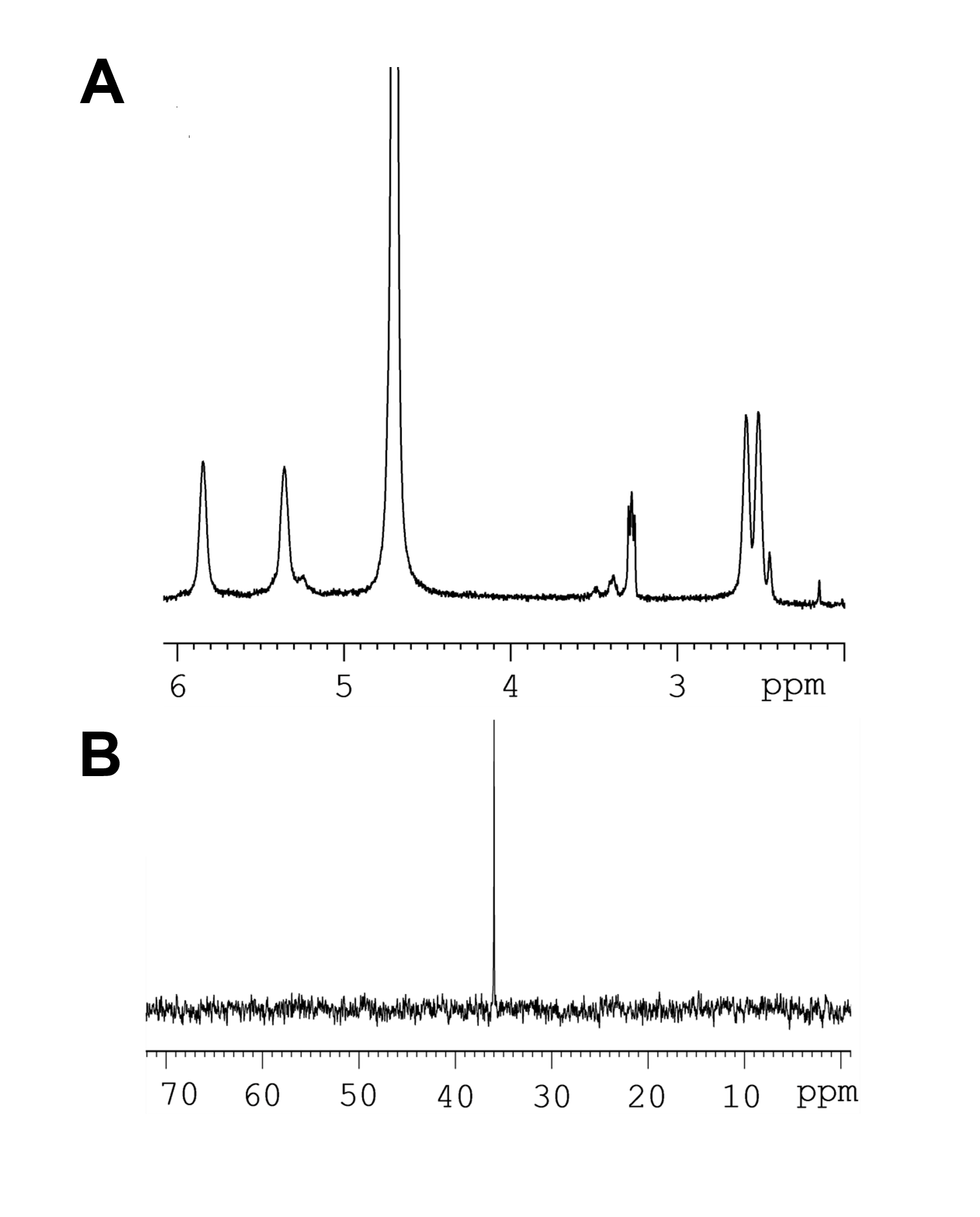
**

**Figure S7**. NMR characterization of Pt-BP complex. ^1^H NMR (A) and ^31^P NMR (B) of Pt-BP complex in D_2_O.

**Table S1.** Characteristics of the radionuclide purity of ^195m^Pt(NO_3_)_2_(en)

| Nuclide | Activity [Bq] | Percentage [%] |
| --- | --- | --- |
| Pt-195m | 1,40E+07 | 96.3 |
| Pt-197 | 4,14E+05 | 2.8 |
| Pt-191 | 2,39E+03 | 0.02 |
| Au-198 | 4,03E+03 | 0.03 |
| Au-199 | 1,10E+05 | 0.8 |
| Ir-192 | 4,58E+03 | 0.03 |
| Ir-194 | 7,52E+03 | 0.05 |

References:

1 Iafisco, M. *et al.* Smart delivery of antitumoral platinum complexes from biomimetic hydroxyapatite nanocrystals. *Journal of Materials Chemistry* **19**, doi:10.1039/b914379c (2009).

2 Margiotta, N. *et al.* Bisphosphonate complexation and calcium doping in silica xerogels as a combined strategy for local and controlled release of active platinum antitumor compounds. *Dalton Trans*, 3131-3139, doi:10.1039/b705239a (2007).

3 Neri, D. & Supuran, C. T. Interfering with pH regulation in tumours as a therapeutic strategy. *Nat Rev Drug Discov* **10**, 767-777, doi:10.1038/nrd3554 (2011).

4 Parks, S. K., Chiche, J. & Pouyssegur, J. Disrupting proton dynamics and energy metabolism for cancer therapy. *Nat Rev Cancer* **13**, 611-623, doi:10.1038/nrc3579 (2013).

5 Farbod, K. *The use of bisphosphonates for bone-specific drug delivery*, Radboud University Medical Center, (2016).

6 Pasini, A., Caldiroia, C., Spinelli, S. & Valsecchi, M. Comments on Different Synthetic Methods for the Preparation of Diammine and bis(Amine) Organodicarboxylatoplatinum(II) Complexes. *Synthesis and Reactivity in Inorganic and Metal-Organic Chemistry* **23**, 1021-1060, doi:10.1080/15533179308016879 (1993).
